# A novel method of swin transformer with time-frequency characteristics for ECG-based arrhythmia detection

**DOI:** 10.3389/fcvm.2024.1401143

**Published:** 2024-06-07

**Authors:** Siyuan Chen, Hao Wang, Huijie Zhang, Cailiang Peng, Yang Li, Bing Wang

**Affiliations:** ^1^ Heilongjiang University of Chinese Medicine, Harbin, China; ^2^ College of Computer Science and Technology, Harbin Engineering University, Harbin, China; ^3^ First Affiliated Hospital, Heilongjiang University of Chinese Medicine, Harbin, China

**Keywords:** electrocardiogram, deep learning, arrhythmia, wavelet time-frequency map, swin transformer

## Abstract

**Introduction:**

Arrhythmia is an important indication of underlying cardiovascular diseases (CVD) and is prevalent worldwide. Accurate diagnosis of arrhythmia is crucial for timely and effective treatment. Electrocardiogram (ECG) plays a key role in the diagnosis of arrhythmia. With the continuous development of deep learning and machine learning processes in the clinical field, ECG processing algorithms have significantly advanced the field with timely and accurate diagnosis of arrhythmia.

**Methods:**

In this study, we combined the wavelet time-frequency maps with the novel Swin Transformer deep learning model for the automatic detection of cardiac arrhythmias. In specific practice, we used the MIT-BIH arrhythmia dataset, and to improve the signal quality, we removed the high-frequency noise, artifacts, electromyographic noise and respiratory motion effects in the ECG signals by the wavelet thresholding method; we used the complex Morlet wavelet for the feature extraction, and plotted wavelet time-frequency maps to visualise the time-frequency information of the ECG; we introduced the Swin Transformer model for classification and achieve high classification accuracy of ECG signals through hierarchical construction and self attention mechanism, and combines windowed multi-head self-attention (W-MSA) and shifted window-based multi-head self-attention (SW-MSA) to comprehensively utilise the local and global information.

**Results:**

To enhance the confidence of the experimental results, we evaluated the performance using intra-patient and inter-patient paradigm analyses, and the model classification accuracies reached 99.34% and 98.37%, respectively, which are better than the currently available detection methods.

**Discussion:**

The results reveal that our proposed method is superior to currently available methods for detecting arrhythmia ECG. This provides a new idea for ECG based arrhythmia diagnosis.

## 1 Introduction

Currently, cardiovascular diseases (CVD) exhibit the highest morbidity and mortality rates worldwide, posing a serious threat to human health (1). According to the World Health Organization (WHO), CVD-associated deaths account for approximately 32% of the total number of deaths each year (2). Arrhythmia, a phenomenon that causes the heart to beat irregularly due to abnormal functioning of the heart's electrical system, is one of the major manifestations of underlying CVD (3). In many cases, arrhythmia patients often remain asymptomatic and later lead to diseases that cannot be easily diagnosed, resulting in serious consequences, for example, heart failure, stroke, and even sudden cardiac death (4, 5). Therefore, accurate and rapid detection of arrhythmia is particularly important for better treatment outcomes and long-term survival of the patient. Therefore, detecting arrhythmia at an early stage can minimize the chances of life-threatening situations in the future.

Currently, there are several diagnostic methods for arrhythmia, such as electrocardiogram (ECG), cardiovascular magnetic resonance imaging (MRI), and cardiac computed tomography (CT) (6–8). As an objective indicator of the occurrence, propagation, and recovery process of cardiac excitation, ECG plays a crucial role in the diagnosis of heart diseases. Moreover, due to the non-invasive procedure and low-cost advantages, ECG is most commonly used for detecting arrhythmia in clinical practice.

ECG data are usually affected by multiple factors, and data preprocessing can improve the data quality. Noise reduction is widely used as a common method for preprocessing. Sharma et al. (9) proposed noise reduction based on eigenvalue decomposition of Hankel matrix, which achieved better performance. Zhang et al. (10) proposed noise reduction of ECG signals by using coif3 wavelet and trap filter, which improves the accuracy of extracted ECG parameters by suppressing the noise of P-wave and T-wave. Among many noise reduction methods, wavelet thresholding is suitable for various types of signal noise reduction, including biomedical signals, images, audio, etc., so it has a wide range of application prospects (11–13). Therefore, in this paper, wavelet thresholding method is used to achieve ECG noise reduction.

Feature extraction is an important component of machine-learning-based ECG diagnosis. Currently, feature extraction processes have been optimized based on the time and frequency domain aspects of the ECG (14, 15). Time domain feature extraction mainly captures the dynamic characteristics of the signal with respect to time but this feature has limitations in dealing with non-stationary signals. Frequency domain feature extraction can reveal the frequency of components and spectral features of the ECG, but transient information may be omitted when extracting the signal. In contrast, time-frequency domain feature extraction combines both time and frequency domain information, making the signal analysis more comprehensive by capturing the dynamic changes and frequency characteristics of the ECG. Currently, ECG feature extraction based on time-frequency features has become more and more popular, Qurraie et al. (16) extracted time-frequency features and statistical features of ECG signals along the RR intervals for arrhythmia classification, and Sharma et al. (17) proposed time-frequency matrix-based modified features for detecting coronary artery disease (CAD), which have all achieved good results. Wavelet time-frequency maps can visualise the time-frequency characteristics of the signal and have significant advantages in feature extraction (18). Therefore, this study primarily focuses on the time-frequency domain and adopts the visualized wavelet time-frequency diagram to represent ECG features. This combined approach can effectively extract almost all ECG time-frequency domain features and provide a guarantee for the subsequent accurate classification work.

With the continuous development of computer-aided technologies in the medical field, the number of research works on the ECG detection method is gradually increasing. Diker et al. (19) used the Pan-Tompkins algorithm and the discrete wavelet transform (DWT) to extract the key points of ECG signals for ECG classification, and improved the wavelet kernel limit learning machine to determine the wavelet coefficients. Shirin et al. (20) used electrocardiogram data from three different databases, combined with temporal and spectral analyses and nonlinear dynamics, and were able to efficiently distinguish between ventricular fibrillation (VF) and non-VF arrhythmias and applied B bifurcated decision tree (BDT) and support vector machine (SVM) classifiers for arrhythmia classification. These methods have not only improved the accuracy of cardiovascular disease diagnosis, but also reduced the time required for diagnosis. In recent years, deep learning methods have achieved better results in several fields, including biosignal analysis (21), face recognition (22), computer vision (23), and character recognition (24). In ECG detection, the application of deep learning methods has also become substantially widespread (25–27). Currently, traditional networks such as convolutional neural networks (CNN) and recurrent neural networks (RNN) are most frequently used for arrhythmia diagnosis (28, 29). However, these techniques have several limitations in facing long-range dependencies and dealing with global information capture. The new network architecture of the emerging Transformer (30) shows certain advantages over previous models in the field of deep learning. The Transformer model has been developed considering the weightage of each position over the others through a self-attention mechanism, and positional coding to convey information about the sequence structure. In addition, jump connections between the outputs and inputs of each sublayer have been added to this model. Compared with the traditional model, the Transformer can effectively solve more complex problems involving long-range dependencies and global information capture. Simultaneously, the Transformer can perform parallel computation by calculating the number of dependencies between different positions in the input sequence, which improves the training speed of the model. Developed based on the Transformer model, the Swin Transformer offers specific improvements for computer vision to increase the efficiency of image-specific processing, thus making the Transformer architecture more compatible and efficient for a wide range of large-scale computer vision tasks (31). Therefore, we employed the Swin Transformer model to classify the ECG features.

In this study, we first pre-processed the ECG data from the MIT-BIH arrhythmia database to improve the data quality, then extracted the time-frequency features of the ECG by wavelet time-frequency mapping. Swin Transformer model was used to classify various types of arrhythmia, which in turn enabled the effective detection of cardiac arrhythmia. We found that the Swin Transformer model was effective in prompting early warning and auto-diagnosis of arrhythmia, which provided a new avenue for arrhythmia detection.

## 2 Materials and methods

**Figure 1:**
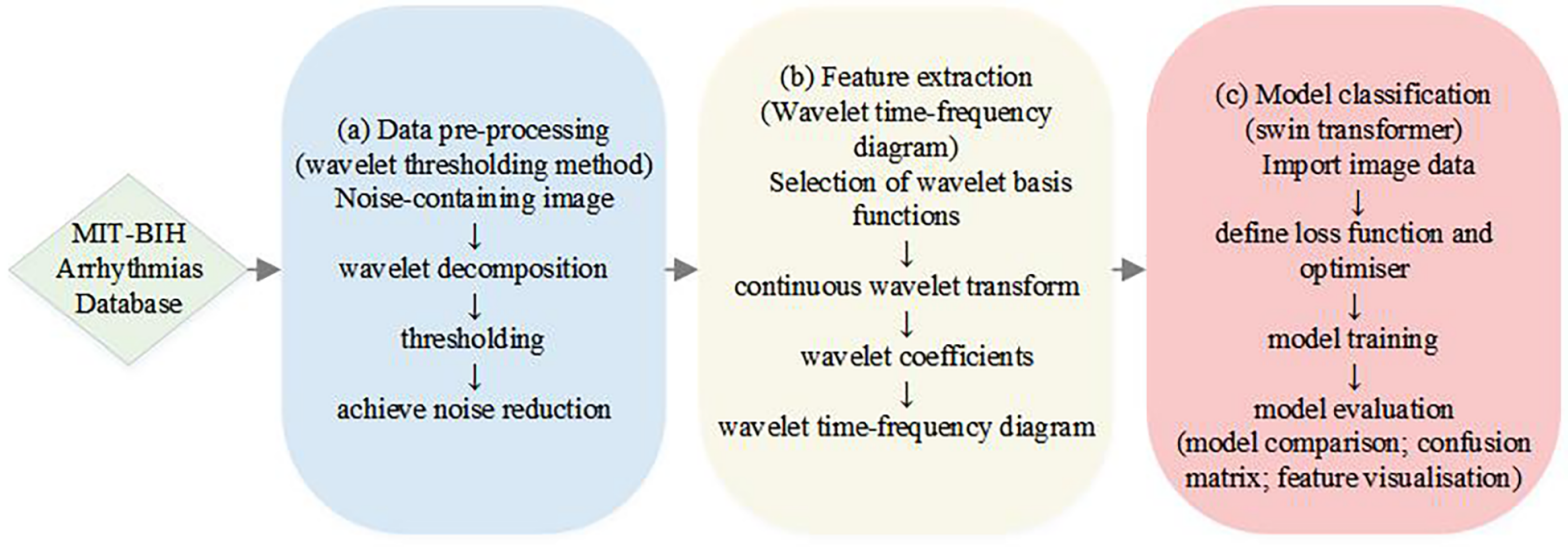
A flowchart illustrating the process of achieving ECG classification of arrhythmias.

Arrhythmia classification has always been an important issue in the field of medicine, which is significant for diagnosis and treatment. With our proposed method, we are able to identify different types of arrhythmias more accurately and provide doctors with a more reliable auxiliary diagnostic tool, which further improves the efficiency and accuracy of patient diagnosis and treatment. In this study, we propose an ECG signal processing and classification method based on the wavelet threshold method and the Swin Transformer model, which has the potential for accurate ECG data analysis and classification in clinical applications. The overall flowchart of the experiments in this study is shown in Figure 1, which consists of three main parts. The first part is the preprocessing of the data, which introduces the new application of wavelet thresholding method in ECG data denoising to remove the effects of high frequency noise, artefacts, electromyographic noise and respiratory motion produced on ECG. We perform wavelet transform on the original ECG signal, which makes the signal decomposed into wavelet components with different scales and frequencies, and choose the soft thresholding method for thresholding, which improves the quality of ECG. The second part is to complete the feature extraction of ECG data, we adopt the complex Morlet wavelet as the wavelet basis function, and then the continuous wavelet transform(CWT) is used to capture the feature information of the signal in time and frequency simultaneously, and the wavelet time-frequency diagram is drawn to show the ECG features, so as to achieve a more intuitive presentation of the time-frequency information of ECG, and to improve the accuracy and reliability of the analysis of ECG signals. The third part is to introduce the Swin Transformer model for ECG classification, which uses a hierarchical construction method similar to CNN, reduces the computational complexity by calculating the self-attention within the window, and achieves window-to-window information transfer by moving window, which has a good accuracy in clinical diagnosis. We input the wavelet time-frequency maps into the Patch Partition module for chunking, sampling through the Patch Merging layer, and then construct feature maps of different sizes through four stages, and in order to combine local and global information, we use windowed multi-head self-attention (W-MSA) and shifted window-based multi-head self-attention (SW-MSA) in pairs. For each sample, the features are standardized, the mean and variance of the features are calculated, and the feature map is pooled along the spatial dimension. The globally pooled feature vector is used as input, and the output of the last fully connected layer is used as the final output of the model. Cross entropy loss is used as the loss function, and the Adam optimizer is selected for model optimization to achieve signal classification. This model has good accuracy in clinical diagnosis. Finally, model evaluation is achieved through model comparison confusion matrix and feature visualization.

### 2.1 Dataset

The MIT-BIH Arrhythmia database, created in collaboration between the Massachusetts Institute of Technology (MIT) and Beth Israel Hospital (BIH), is one of the most widely used resources in the field of arrhythmia detection and classification (32). The dataset covers many types of arrhythmias, including supraventricular and ventricular premature beats, atrial fibrillation, and atrial flutter.

In this study, we used the MIT-BIH Arrhythmia database for the classification of arrhythmias. This dataset included 48 ECG recordings from 47 volunteers, each of which lasted 30 min and contained more than 116,000 heartbeats, consisting of two signal channels V and II, and recorded at a sampling rate of 360 Hz and 11-bit resolution with a range of 10 mV (33–35).

In this experiment, we randomly divided the MIT-BIH Arrhythmia database into ten subsets, using nine folds of data as the training set and the remaining one fold as the validation set. The process was repeated ten times, choosing a different validation fold each time. During each validation process, the model is trained using the currently selected training set and the model performance is evaluated on the corresponding validation set. The accuracy, precision, sensitivity, specificity, F1 score and AUC values were recorded for each validation. Afterwards, the above metrics of each of the ten validations are averaged to obtain the final model evaluation results.

### 2.2 Pre-processing

Usually, the ECG recording includes artefactual contaminations, which incorrectly extract the local waveform of the ECG. Therefore, performing pre-processing is an essential step toward a precise and automatic ECG classification. Pre-processing of ECG data includes de-baseline drifting, filtering, noise reduction, and heartbeat detection, aiming to improve the quality of ECG signals (36). Among them, noise reduction is the most important step in experimental pre-processing. In this work, we used the wavelet thresholding method (37) to denoise the raw data, in order to remove the effects of high-frequency noise, artifacts, electromyographic noise, and respiratory movements on ECG. First, the db6 wavelet was used to decompose the signal into levels 1–3, and subsequently, decomposed signals were adjusted to the baseline using the rigrsure soft threshold selection method. Compared with the hard thresholding method, the soft thresholding method improves the defect of discontinuity at the threshold point, so smoother data are obtained in the soft thresholding process. The threshold processing formula of the soft thresholding method is expressed as Equation (1):

(1) $$\hat{w}=\{\begin{array}{c} {}[\mathrm{sgn}(w)](|w|-T),|w|\geq T\\ 0,|w|<T \end{array}$$

where *w* is the decomposed wavelet coefficient, $\hat{w}$ is the thresholded wavelet coefficient, and *T* denotes the threshold function.

For healthcare professionals to accurately identify and understand the ECG features, the Association for the Advancement of Medical Instruments (AAMI) has classified the MIT-BIH arrhythmia database into five AAMI heartbeat categories: *N* for normal beats, *S* for supraventricular abnormalities, *V* for ventricular abnormalities, *F* for fusion beats, and *Q* for unclassified beats (38, 39).

### 2.3 ECG feature extraction

The wavelet time-frequency diagram is one of the important methods to extract the time-frequency features of ECG, which converts one-dimensional signals into a two-dimensional time-frequency image through the CWT. Thus, the wavelet time-frequency diagram could be helpful to further understand the essential properties of ECG, and enhance the analysis and diagnosis of diseases (40, 41).

Here, we used the complex Morlet wavelet for the wavelet analysis, which is expressed as Equation (2):

(2) $$\psi (t)=(\pi F_{b}{)}^{0.5}e^{2i_{\pi }F_{c}t}e^{-t^{2}/F_{b}}$$

where *F_b_* denotes the bandwidth factor.

The wavelet coefficient of different scales and frequency bands of wavelet components that have been decomposed in the pre-processing step are obtained by wavelet transformation that gives different signal resolutions at different time-frequency characteristics, and the formula for the CWT is expressed as Equation (3):

(3) $$U(\alpha ,\beta )=\overset{+\infty }{\underset{-\infty }{\int }}x(t)\bar{\psi (t)}dt=\frac{1}{\sqrt{\left|\alpha \right|}}\overset{+\infty }{\underset{-\infty }{\int }}x(t)\bar{\psi \left(\frac{t-\beta }{\alpha }\right)}dt$$

where U(α,β) denotes the coefficient of the wavelet function; α and β denotes the scaling and translation factors; x(t) denotes the original signal; ψ(t) indicates the wavelet basis function; and $\bar{\psi (t)}$ refers to the conjugate complex of ψ(t). These wavelets are generated by the mother wavelet ψ scaling and translation.

Following are the steps for calculating the time-frequency diagram of the wavelet components:

The actual frequency *F_a_* corresponding to the scaling factor is denoted as Equation (4):

(4) $$F_{a}=F_{c}\cdot \frac{\;f_{s}}{\alpha }$$

where *F_c_* denotes the wavelet center frequency factor in Hz, and *f_s_* denotes the sampling frequency.

The scale series *t* is expressed in the following form so that the changed frequency series is presented as an isotropic series *A*:

(5) A={c/totalscal,⋯,c/(totalscal−1),c/4,c/2,c}

(6) $$c=2F_{c}\cdot \;totalscal\;$$

where the length of the scale sequence *totalscal* is set to 256, and *c* is a constant.

Substituting the expression of Equation (6) into Equation (5), we can obtain the desired scale sequence. Through the scale sequence and wavelet basis, the wavelet coefficient matrix can be derived from Equation (3), and the wavelet time-frequency diagram of the original vibration signal can be constructed by combining the time series and the actual frequency series.

### 2.4 ECG classification model

The Swin Transformer network model is a deep learning model proposed by Microsoft Research Asia in 2021. This model uses a hierarchical structure to extract features by calculating attention within each window (31). In this study, we applied the Swin Transformer network model to segment an image with an input of *h *× *w *× 3 RGB into non-overlapping equal-sized *N* × (4 × 4 × 3) image blocks, where *h* and *w* were the height and width of the input image, respectively, and *N* was the effective length of the input sequence for the 31model.

When the linear embedding layer projects a tensor with feature dimension *h*/4 × *w*/4 × 48 to any dimension *C*, the feature dimension at this point becomes *h*/4 × *w*/4 × *C*. Hence, we first performed layer normalization (LN) by passing the image block sequence into two consecutive Swin Transformer blocks, and then conducted W-MSA analysis. The W-MSA divides the input sequence into different windows based on MSA to reduce the complexity of the calculation. Attention is calculated by extracting the correlation between local patches in the window. The basic formula of MSA can be expressed as Equation (7):

(7) $$Attention\;(Q,K,V)=softmax\;\left(\frac{QK^{T}}{\sqrt{d_{k}}}\right)V$$

Where *Q*, *K* and *V* denote the Query, Key and Value matrices, respectively; *softmax* denotes the weight assigned to all the Keys of each Query; *d_k_* denotes the dimension of each element in the *K* matrix and applies weights to the Value matrix.

Assuming that the size of each local block is m_*_n and the dimension of the transformed matrix is Z_*_Z, the computation of MSA can be done by Equation (8):

(8) $$\Omega (MSA)=4mnZ^{2}+2(mn{)}^{2}Z$$

Assuming that a graph is divided into H*H local blocks, the computation of W-MSA can be estimated as Equation (9):

(9) $$\Omega (WMSA)=4mnZ^{2}+2H^{2}mn^{2}Z$$

Next, *LN* and multilayer perception (*MLP*) are performed to train the model with features at a deeper level, and this is the end of the first module. Subsequently, SW-MSA based on moving windows is carried out to realize mutual communication between windows, where W-MSA and SW-MSA are used in pairs, and their module calculations can be performed using Equation (10):

(10) $$\begin{array}{rl} & {\hat{F}}^{l}=WMSA(LN(F^{l-1}))+F^{l-1}\\ & F^{l}=MLP(LN({\hat{F}}^{l}))+{\hat{F}}^{l}\\ & {\hat{F}}^{l+1}=SW\text{-}\;MSA(LN(F^{l}))+F^{l}\\ & F^{l+1}=MLP(LN({\hat{F}}^{l+1}))+{\hat{F}}^{l+1} \end{array}$$

where ${\hat{F}}^{l}$ and $F^{l}$ denote the output features of the (S)W-MSA module and the MLP module, respectively.

Finally, the output result is obtained by MLP, and the number of output sequences is the same as that of the input. In the second stage, the adjacent 2 × 2 image blocks are stitched together by merging layers, and the output of the feature dimension after feature conversion is *h*/8 × *w*/8 × 2*C*. The process is repeated, and the eigendimensions of stages 3 and 4, respectively, give output as *h*/16 × *w*/16 × 4*C* and *h*/32 × *w*/32 × 8*C*.

After completing the execution of the Swin Transformer block, the final ECG classification results are generated through normalization, global pooling, and full connection.

## 3 Results

### 3.1 Eigenvalue analysis

**Figure 2:**
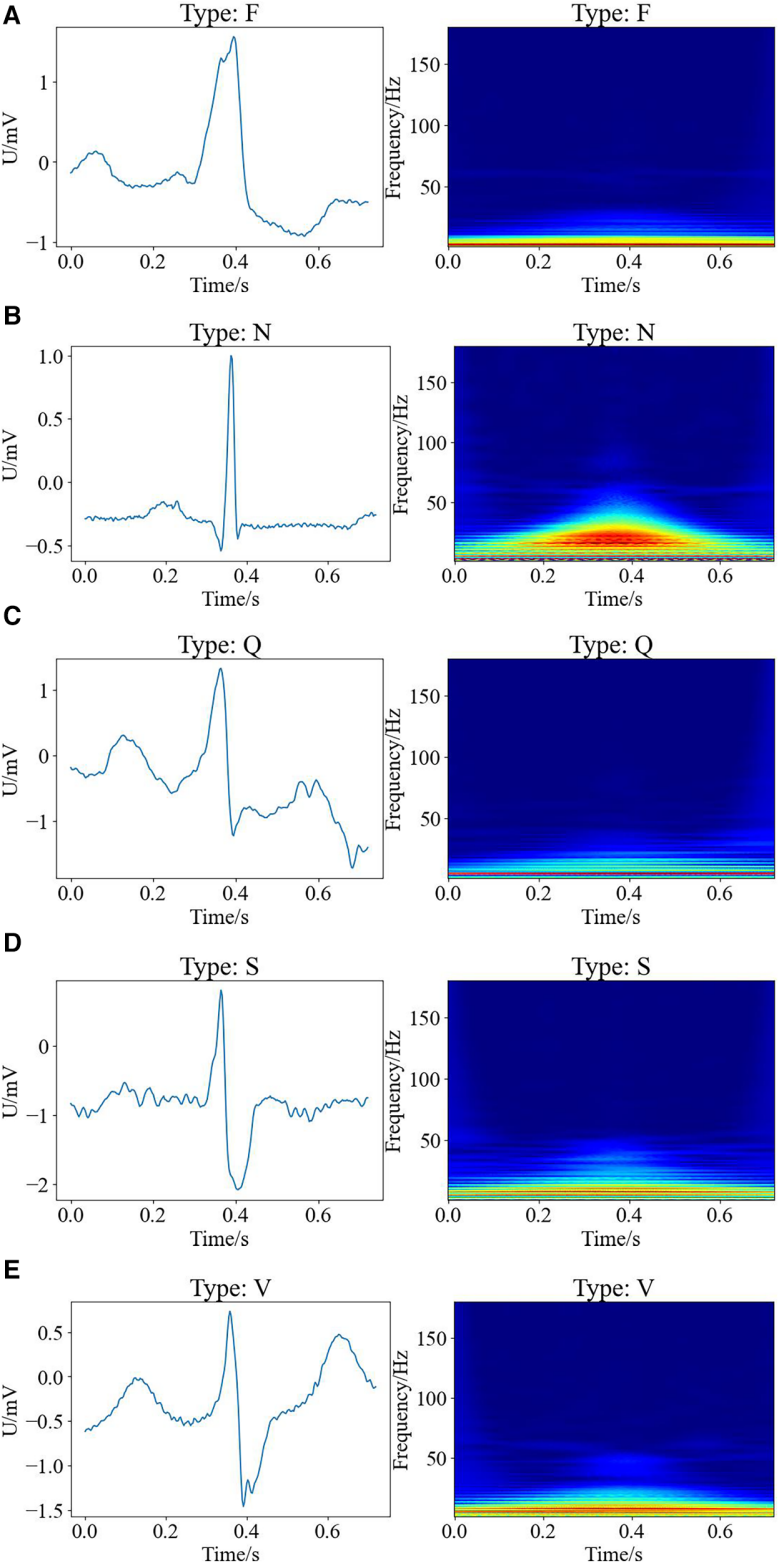
Raw signal maps and wavelet time-frequency maps of different types of arrhythmias, with raw signal maps on the left and corresponding wavelet time-frequency maps on the right. (**A**) F type; (**B**) N type; (**C**) Q type; (**D**) S type; (**E**) V type.

Here, we exploited wavelet time-frequency maps to transform one-dimensional ECG into two-dimensional images with time-frequency features, thus presenting ECG results in a better way, where the warm and cold colors of the wavelet time-frequency maps indicated the wavelet energy values of the signals in the time-frequency domain. The warm color represented the high-energy regions, while the cold color referred to the low-energy regions, and the horizontal and vertical axes of the images indicated the time and frequency, respectively. Figure 2 shows the raw signal maps of the five types of arrhythmias and the wavelet time-frequency maps presented in the time-frequency domain.

It can be observed that there are differences between the wavelet time-frequency maps corresponding to different types of cardiac beats. Different colors present an irregular block-like distribution, with the warm color area of the N-shaped heart beat being significantly larger than other types, and having the largest range and higher energy. The cold color area of Q-shaped heart beats is significantly more pronounced than other types. Compared to the S-shaped heart beat, the V-shaped heart beat exhibits a brighter overall color and a larger range. Additionally, the color range of F-type heart beats is the smallest compared to other types, indicating that the color distribution of wavelet time-frequency maps can effectively characterize significant differences between different types of arrhythmias. Therefore, wavelet time-frequency maps have the potential to serve as evaluation indicators for different types of ECG abnormalities. In this work, we extracted the time-frequency features of ECG data for different types of arrhythmias through wavelet time-frequency maps, laying the foundation for subsequent classification tasks.

### 3.2 Performance evaluation

We utilized 10-fold cross-validation to compare the performance of our proposed model with other commonly used models for arrhythmia detection. To enhance the credibility and robustness of our findings, we conducted intra-patient and inter-patient paradigm analyses, respectively. Intra-patient analyses evaluated the model's ability to track heart rate variability by partitioning the database into 10 groups, with one group serving as the test set and the remainder as the training set in each iteration. Table 1 illustrates an example of within-patient analysis, showcasing the specific performance of 8 different methods for arrhythmia detection at 95% confidence intervals. The evaluated metrics include accuracy, precision, sensitivity, specificity, F1 score, and AUC. Through the calculation of these confidence intervals in 10-fold cross-validation, we were able to assess the stability and variability of the models across different subsets of random data. This approach aids in identifying and addressing potential overfitting issues and ensures the model's robust generalization capabilities.

**Table 1:** Performance comparison between our model and previously reported models for arrhythmia detection in intra-patient paradigm. (unit: %).

Methodology	Accuracy (CI)	Precision (CI)	Sensitivity (CI)	Specificity (CI)	F1-Score (CI)	AUC (CI)
1D-CNN (42)	91.28 (89.49, 93.07)	93.58 (91.79, 95.37)	89.29 (87.50, 91.08)	92.83 (91.04, 94.62)	90.51 (88.72, 92.30)	93.27 (91.48, 95.06)
1D-CNN (28)	97.19 (94.93, 99.45)	95.62 (93.36, 97.88)	96.17 (93.91, 98.43)	91.73 (89.47, 93.99)	97.81 (95.55, 99.07)	96.51 (94.25, 98.77)
CNN-LSTM (43)	97.93 (96.86, 98.99)	95.37 (94.30, 96.44)	98.04 (96.97, 99.11)	97.62 (96.55, 98.69)	97.95 (96.88, 99.02)	97.38 (96.31, 98.45)
2D-CNN (42)	98.74 (97.10, 99.38)	97.69 (96.05, 99.33)	99.08 (98.44, 99.52)	94.82 (93.18, 96.46)	98.63 (97.99, 99.27)	98.14 (96.50, 99.78)
2D-CNN (44)	98.97 (98.83, 99.11)	94.24 (92.10, 96.38)	96.38 (94.24, 98.52)	99.53 (98.39, 99.67)	96.72 (94.58, 98.86)	98.95 (97.81, 99.08)
DBB, AdaBoos (45)	99.04 (98.25, 99.83)	98.37 (97.58, 99.16)	97.52 (96.73, 98.31)	99.31 (98.52, 99.47)	98.84 (98.05, 99.63)	97.62 (96.83, 98.41)
ECG DETR (46)	99.19 (98.56, 99.82)	97.91 (97.28, 98.54)	98.14 (97.51, 98.77)	99.27 (98.64, 99.90)	98.31 (97.68, 98.94)	99.08 (98.45, 99.71)
**Proposed**	**99.34** (**98.99, 99.69)**	**98.71** (**98.36, 99.06)**	**99.49** (**99.14, 99.84)**	**99.57** (**99.22, 99.92)**	**98.96** (**98.61, 99.31)**	**99.35** (**99.00, 99.78)**

CI: 95% confidence intervals (Lower–Upper bound).

The bold values represent the specific performance values of our proposed method.

However, for clinical applications, the training and testing sets will not be sourced from the same patient. To address this challenge, we conducted an inter-patient paradigm analysis. We divided the 47 subjects into 10 groups. Each time, we randomly selected one group and used their ECG data with different labels as the test set, while the remaining ECG datasets were used as the training set. In other words, the same subject cannot be part of both the training and testing datasets simultaneously. We trained the model using the above-described data and repeated this process ten times, selecting different validation folds each time. Finally, we obtained the average performance indicators, as shown in Table 2.

**Table 2:** Performance comparison between our model and previously reported models for arrhythmia detection in inter-patient paradigm. (unit: %).

Methodology	Accuracy (CI)	Precision (CI)	Sensitivity (CI)	Specificity (CI)	F1-Score (CI)	AUC (CI)
1D-CNN (42)	89.92 (88.10, 91.74)	90.89 (89.07, 92.71)	87.68 (85.86, 89.52)	91.77 (89.95, 93.59)	87.61 (85.79, 89.43)	90.58 (88.76, 92.48)
1D-CNN (28)	94.48 (92.30, 96.66)	93.62 (91.44, 95.82)	94.35 (92.17, 96.53)	89.49 (87.31, 91.67)	95.44 (93.26, 97.62)	93.14 (90.96, 95.32)
CNN-LSTM (43)	96.54 (94.97, 98.11)	92.66 (91.09, 94.23)	95.12 (93.55, 96.69)	96.49 (94.92, 98.06)	96.42 (94.85, 97.99)	95.82 (94.25, 97.39)
2D-CNN (42)	97.28 (95.30, 99.26)	95.55 (93.57, 97.53)	97.93 (95.95, 99.91)	93.06 (91.08, 95.04)	97.01 (95.03, 98.99)	94.37 (92.39, 96.35)
2D-CNN (44)	97.74 (96.20, 99.28)	91.82 (89.28, 94.36)	94.44 (91.90, 96.98)	98.23 (95.69, 98.77)	93.95 (91.41, 96.49)	95.25 (92.71, 97.79)
DBB, AdaBoos (45)	96.73 (95.74, 97.72)	97.31 (96.32, 98.32)	96.14 (95.15, 97.13)	96.56 (95.57, 97.55)	95.98 (94.99, 96.97)	94.55 (93.56, 95.54)
ECG DETR (46)	97.25 (96.18, 98.32)	95.22 (94.15, 96.29)	96.29 (95.22, 97.36)	98.14 (97.07, 99.21)	97.18 (96.11, 98.25)	97.38 (96.31, 98.45)
Proposed	**98.37** (**97.75, 98.99)**	**96.64** (**96.02, 97.26)**	**97.20** (**96.58, 97.82)**	**97.12** (**96.50, 97.74)**	**97.64** (**97.02, 98.26)**	**97.68** (**97.06, 98.30)**

CI: 95% confidence intervals (Lower–Upper bound).

The bold values represent the specific performance values of our proposed method.

Compared to the performance records of different methods in detecting arrhythmia, our model achieved classification accuracies of 99.34% and 98.37% in intra-patient and inter-patient case analyses, respectively. These values are 0.15% and 0.63% higher than the current best accuracy achieved by an arrhythmia classification model. Additionally, the classification accuracy of our model reached 98.71% and 96.64%, with sensitivity values of 99.49% and 97.20%, specificity values of 99.57% and 97.12%, F1 scores of 98.96% and 97.64%, and AUC values of 99.35% and 97.68%, respectively. The AUC value (47) provides an overall measure of the model's performance across all possible classification thresholds, so we have included this indicator in Tables 1, 2. The high AUC value of our proposed method demonstrates its strong ability to distinguish between different classes. In summary, our model exhibits superior performance compared to commonly used arrhythmia classification models.

Despite promising results of previous studies in classifying arrhythmias, long-distance dependency remains a major challenge in precise modeling for arrhythmia classification. However, the present study demonstrated improved performances of the model by adopting an advanced model architecture that comprehensively captured long-distance dependencies in ECG.

**Figure 3:**
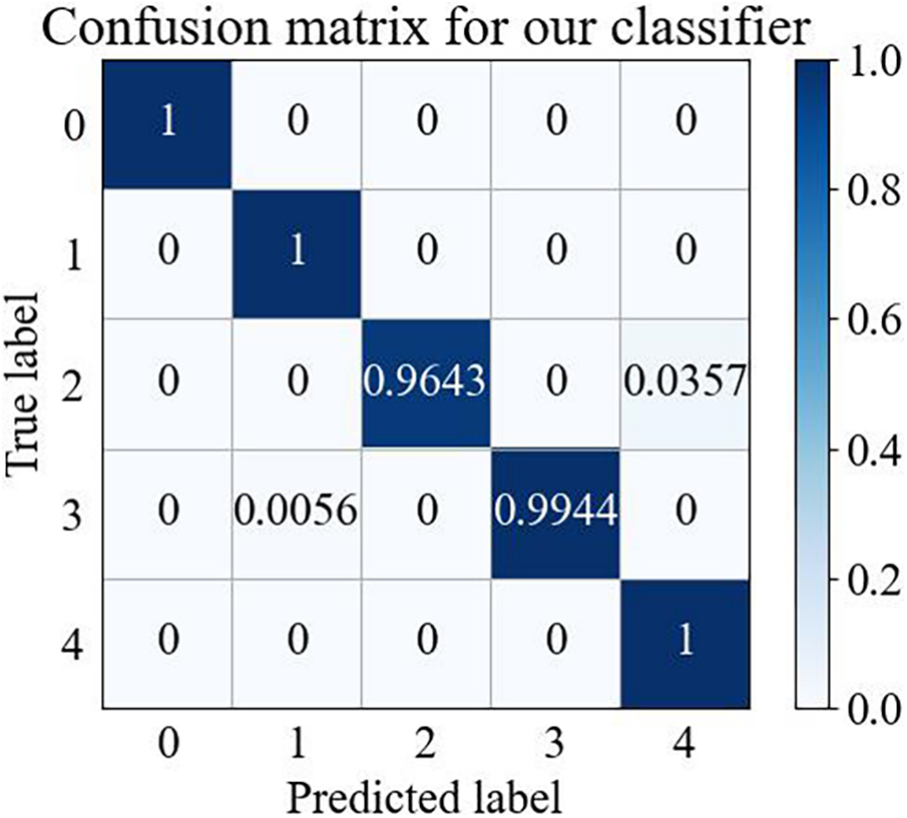
Wavelet time-frequency diagram-swin transformer confusion matrix.

The confusion matrix is one of the most important methods of evaluating the performance of a classifier, and the confusion matrix for five types of arrhythmias for the test set is illustrated in Figure 3.

Figure 3 shows that the wavelet time-frequency map combined with the Swin Transformer model has a better recognition effect on different arrhythmia states, thus providing reliable support in arrhythmia detection. This result further confirmed that our model could achieve effective classification of arrhythmia and demonstrated the potential of practical applications of this model in the medical field.

### 3.3 Feature visualization

**Figure 4:**
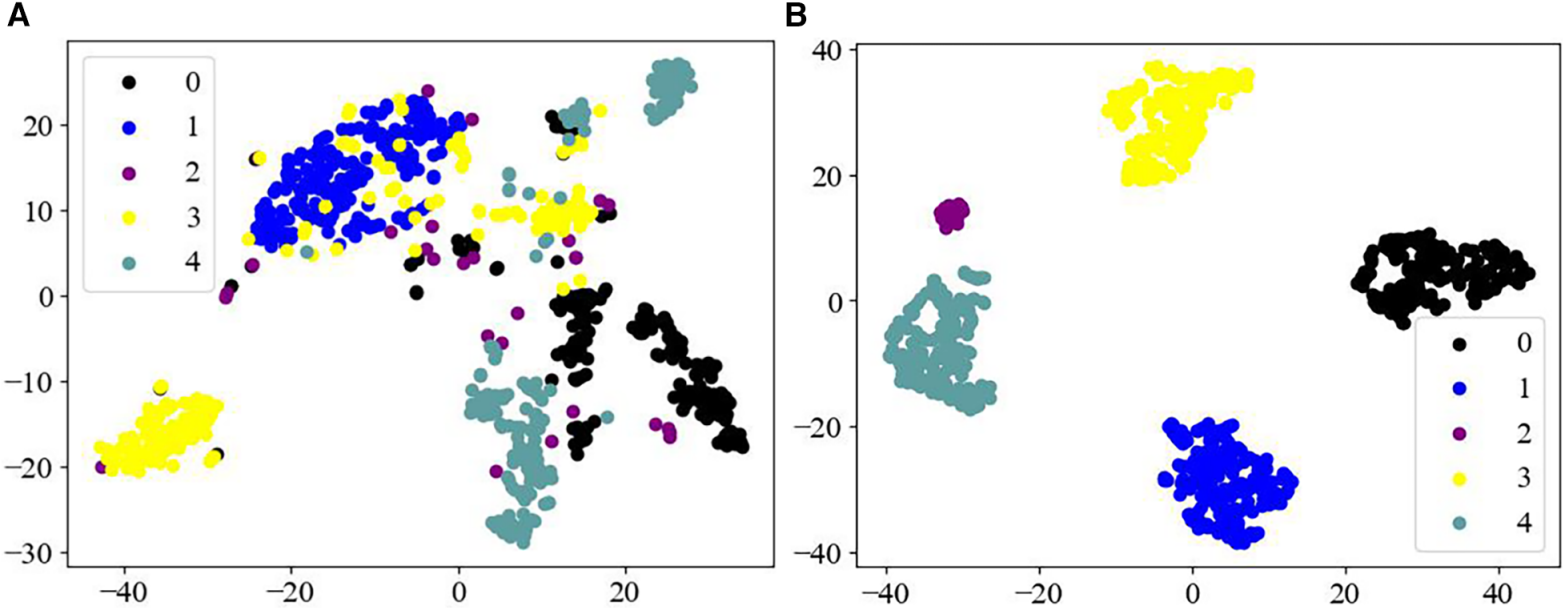
Feature visualization map. (**A**) Raw data of different categories; (**B**) features of different categories as output from the fully connected layers of the model.

In this study, we used the t-distributed stochastic neighbor embedding (T-SNE) method (48) to visualize the extracted multidimensional ECG features in a two-dimensional space (Figure 4). T-SNE is a powerful downscaling and visualization tool for transforming dimensions. It achieves visualization mainly through iterative optimization of the position of high-dimensional data points in the low-dimensional space, to generate closely spaced similar data points in the low-dimensional space.

Figure 4A displays that the raw data of the MIT-BIH arrhythmia dataset has a high degree of imbalance, and the data are arranged haphazardly, with a large number of overlapping signals in different categories, thus presenting a confusing state. Whereas Figure 4B, as the processed ECG data, demonstrates good separability, with five distinct categories of arrhythmias, achieving an effective output for the different categories of the dataset. Therefore, these observations further indicate that the Swin Transformer model may have a reliable classification ability for different forms of ECG datasets.

### 3.4 Time complexity

CWT is a highly suitable method for multi-scale time-frequency analysis of signals. CWT analyzes signal characteristics at various scales through wavelet transform, offering detailed scale and translation steps, thus enhancing signal processing detail. For each scale, CWT traverses the entire signal and performs convolution operations, with a time complexity of approximately O(N^2^), where N is the signal length. While this method demands significant computation, it yields a highly accurate representation of time and frequency, rendering it particularly suitable for the detailed demands of heart rate anomaly detection.

The Swin Transformer reduces complexity by constraining the computation of the self-attention mechanism to local windows, with a complexity of O(w^2^ × d) within each window, where w is the size of the window and d is the feature dimension. Additionally, through a hierarchical window merging strategy, the Swin Transformer further decreases the global computational complexity to nearly O[N × log(N)]. This structural optimization makes the Swin Transformer efficient and highly expressive when processing large-scale data.

In summary, our method combines the detailed time-frequency analysis of CWT with the efficient data processing capabilities of the Swin Transformer, resulting in more accurate and detailed anomaly detection capabilities. We believe that, for heart rate anomaly detection applications requiring high-precision diagnosis, the associated computational cost is reasonable.

## 4 Discussion

### 4.1 Comparison with other feature extraction

ECG feature extraction refers to extracting the key information from the original ECG waveform. The high-dimensional time series data are then transformed into more resolved, simple, and representative low-dimensional features, which not only facilitate the early detection and diagnosis of diseases but also help observe the development of diseases and better understand the disease mechanism. The most common method of ECG feature extraction involves extracting either the time domain or frequency domain of ECG for research purposes.

Time domain analysis of ECG mainly includes measurement and analysis of RR intervals, P and QRS wave clusters, T wave duration, ST segments, and QT intervals. Alotaiby et al. (14) first pre-processed ECG data from the PTB database by detrending and inversion. Then, to construct feature vectors, the pre-processed ECG data were segmented and 11 statistical features were extracted from each segment. The median and mean values were used to describe the concentration trend of the ECG. While the standard deviation, range, and quartiles were utilized to measure the degree of dispersion of the ECG. By analyzing the kurtosis and skewness of the ECG, a deeper understanding of the amplitude distribution characteristics as well as the symmetry of the signals, can be obtained to facilitate further analysis of the ECG. Extracting ECG features from the time domain aspect has a low computational cost and is relatively simple to implement. However, the method may result in loss of information, rendering the extracted features non-representative (49).

ECG frequency domain analysis involves converting the ECG data from the time domain to the frequency domain by analyzing the ECG spectrum and frequency bands. Merri et al. (15) developed a model to characterize and quantify measurement errors introduced due to limited sampling frequency. The model takes into account the RR interval measurement errors caused by the ECG sampling frequency limitation and calculates the first- and second-order statistics of errors to evaluate the influence of the error on the heart rate variability power spectrum. We found that the error power spectrum had an additional high-pass filter-like term for the heart rate variability power spectrum, revealing the importance of an equilibrium between heart rate variability and the error power spectrum. The limitation of the sampling frequency might introduce errors to the extracted ECG features, suggesting that extracting only the frequency domain features may not accurately represent ECG.

In contrast, time-frequency domain analysis considers both the time and frequency characteristics of the signal within an integrated framework, which provides a more comprehensive understanding of the dynamic nature of the signal and reflects the better performance of the model in analyzing cardiac arrhythmias. Among them, wavelet time-frequency diagram belongs to one kind of time-frequency domain analysis with a wide range of applications in biomedicine, signal processing, and image processing (50–53). Wavelet time-frequency diagram primarily relies on the characteristics of the signal, the selection of a suitable wavelet family as the basis function, and the calculation of the wavelet coefficients. Then the time-frequency diagram is constructed by calculating the amplitude or energy of the coefficients. Given that the wavelet time-frequency map can comprehensively reflect the time-frequency information of the ECG, this study focused on wavelet time-frequency maps to analyze the ECG features of arrhythmia.

### 4.2 Comparison with other classification models

Recent literature investigating the ECG signals using deep learning models is increasing. Kiranyaz et al. (54) proposed an adaptive one-dimensional convolutional neural network (1D-CNN) that could be trained for different patients to achieve an effective detection of ventricular and supraventricular ectopic beats. Based on the CNN, but 1D-CNN has a fixed requirement on the length of the input signal, which can easily lead to information loss. Jangra et al. (55) proposed an updated CNN, called visual geometry group network (VGGNet), to increase the depth and width of the network. Techniques such as small-size convolutional kernel, pooling layer, batch normalization layer, and dropout layer were used to achieve the extraction of more complex features, thereby enhancing the generalization of the model. The VGGNet achieved better results for arrhythmia classification than the CNN alone. Wang et al. (56) used a long short-term memory (LSTM) model to capture temporal information in ECG data to identify abnormalities in arrhythmias, but the model may suffer from gradient vanishing or gradient explosion when dealing with long sequences. Zhang et al. (29) employed an RNN model to learn strong correlations between consecutive ECG signal points and achieved effective classification of ECG signals at different heart rates, but model may be limited in their ability to model long-term dependencies. Jiang et al. (50) proposed a deep neural network model called Multi-Model Multi-Scale Network (MMnet) for more comprehensive analysis of ECG data, but the complexity of the model as well as the computational cost is high. Gokhan et al. (57) utilized a multi-stage classification system, incorporating ECG waveforms and second-order difference plot (SODP) features, along with a deep belief network (DBN) classifier, to successfully distinguish five types of cardiac arrhythmias. In a separate study, they introduced additional techniques such as wavelet packet decomposition, high-order statistics, morphology, and discrete Fourier transform to enhance feature extraction in a multi-class DBN framework (58). The results demonstrated the method's effectiveness in distinguishing a wide range of heartbeats, albeit requiring substantial data and computational training. While these models have achieved promising results, they also have certain limitations, which we summarize in Table 3.

**Table 3:** Comparison of advantages and disadvantages of different models.

Method	Advantage	Disadvantage
1D-CNN (54)	Able to effectively extract local features.	May lead to information loss.
VGGNet (55)	Capable of extracting complex features and exhibiting good generalization ability.	High computational and training costs.
LSTM (56)	Captures long-term dependencies in time series.	May encounter gradient vanishing or exploding issues when dealing with long sequences.
RNN (29)	Captures dynamic feature relationships in ECG signals.	Limited modeling ability for long-term dependencies.
MMnet (50)	Provides comprehensive recording of ECG information.	High model complexity and computational costs.
DBN (57, 58)	Effectively extracts features.	Requires a large amount of data and computational resources.

In common deep learning models, the chain rule is typically used to perform gradient multiplication across multiple layers. If the gradient was less than 1, the chain multiplication led to a decrease in gradient; if the gradient was greater than 1, the chain multiplication led to an increase in gradient. Either decrease or increase in gradients can seriously affect the model's performance, especially in dealing with long-range dependencies. The transformer delayed the process of gradient loss or increment by implementing certain manipulations, such as residual linking and layer normalization, to facilitate an uninterrupted gradient transfer across the layers. Ding et al. (59) used the Transformer-based ECG reduced-dimensional stacked self-encoder model to effectively overcome the long-distance and the long-term dependence problems as well as accurately limit the parallelization during signal processing for detecting arrhythmia. However, Transformer requires high computational and memory costs when facing large-scale images. While Swin Transformer (60), as an emerging deep learning model, has certain advantages in image processing, adopting the windowed self-attention mechanism to partition the image into multiple windows for reducing the computational cost, improving the semantic understanding of the image through global modeling, enhancing the information exchange between the windows by alternately executing the windowed self-attention and translational windowed self-attention, and adapting to different scales of feature extraction by adjusting the size of the window. Swin Transformer combines the advantages of CNN and Transformer in extracting local features and location information, which takes into account both local and global feature extraction capabilities to achieve a precise feature classification. Therefore, in this study, Swin Transformer was chosen to detect arrhythmia, aiming to accurately record ECG information and achieve rapid diagnosis of CVD.

### 4.3 Limitations and future research lines

Although our study demonstrates advancement in developing an arrhythmia detection model through precise classification of ECG data, ECG-related studies are very complex in the sense that the model classifier can also be susceptible to multiple interfering factors such as muscle movement and external noise. There are still certain errors in our model, even after pre-processing the ECG dataset. When applying the classification model, individual differences between different clinical patients should be carefully considered. Therefore, in future studies, we will plan to analyze larger patient ECG datasets, pay more attention to the robustness of the model, test its accuracy, and make it closer to real-world clinical applications.

## 5 Conclusion

In this study, we propose a novel method for detecting arrhythmias using wavelet time-frequency maps and the Swin Transformer. This technology is particularly adept at capturing subtle and transient changes in ECG signals, which are crucial for early arrhythmia diagnosis, especially in asymptomatic patients. Accurate diagnosis facilitates timely interventions by healthcare professionals, enables the development of personalized treatment plans, and ultimately improves patient prognosis and quality of life, highlighting the effectiveness of the Swin Transformer classification model in diagnosing arrhythmias. In our approach, we first employed the wavelet algorithm to denoise the data from the MIT-BIH Arrhythmia Database, thereby enhancing the quality of the original signal. Subsequently, we utilized wavelet time-frequency maps to extract time-frequency features from the ECG signals, effectively representing their distribution. Finally, we applied the Swin Transformer for automatic ECG classification, achieving accuracy rates of 99.34% in intra-patient case analysis and 98.37% in inter-patient case analysis.

Traditional ECG analysis is often limited by time and frequency resolution. However, our proposed new method overcomes this limitation, which is crucial for understanding the complexity of arrhythmias. By combining wavelet time-frequency maps with the Swin Transformer, doctors can analyze electrocardiogram data more accurately and quickly detect arrhythmias, thereby improving diagnostic speed and accuracy. The introduction of the Swin Transformer aims to utilize its advanced self-attention mechanism, which demonstrates excellent performance in processing ECG signals with high spatiotemporal dynamics. Our method achieves high accuracy by analyzing ECG data at multiple frequency and time scales, a crucial aspect in clinical applications as it can provide a more comprehensive diagnostic perspective than traditional methods.

## Data Availability

The original contributions presented in the study are included in the article/Supplementary Material, further inquiries can be directed to the corresponding authors.
